# Can malaria vector control accelerate the interruption of lymphatic filariasis transmission in Africa; capturing a window of opportunity?

**DOI:** 10.1186/1756-3305-6-39

**Published:** 2013-02-22

**Authors:** Louise A Kelly-Hope, David H Molyneux, Moses J Bockarie

**Affiliations:** 1 Centre for Neglected Tropical Diseases, Liverpool School of Tropical Medicine, Pembroke Place, Liverpool L3 5QA, UK

**Keywords:** Lymphatic filariasis, Mass drug administration, Vector control, Long-lasting insecticidal nets, LLINs, Indoor residual spraying, IRS, *Anopheles*, Mosquitoes, Malaria

## Abstract

**Background:**

The Global Programme to Eliminate Lymphatic Filariasis (GPELF) was launched in 2000, and nearly all endemic countries in the Americas, Eastern Mediterranean and Asia-Pacific regions have now initiated the WHO recommended mass drug administration (MDA) campaign to interrupt transmission of the parasite. However, nearly 50% of the LF endemic countries in Africa are yet to implement the GPELF MDA strategy, which does not include vector control. Nevertheless, the recent scale up in insecticide treated /long lasting nets (ITNs/LLINs) and indoor residual spraying (IRS) for malaria control in Africa may significantly impact LF transmission because the parasite is transmitted mainly by *Anopheles* mosquitoes. This study examined the magnitude, geographical extent and potential impact of vector control in the 17 African countries that are yet to or have only recently started MDA.

**Methods:**

National data on mosquito bed nets, ITNs/LLINs and IRS were obtained from published literature, national reports, surveys and datasets from public sources such as Demographic Health Surveys, Malaria Indicator Surveys, Multiple Indicator Cluster Surveys, Malaria Report, Roll Back Malaria and President’s Malaria Initiative websites. The type, number and distribution of interventions were summarised and mapped at sub-national level. and compared with known or potential LF distributions, and those which may be co-endemic with *Loa loa* and MDA is contraindicated.

**Results:**

Analyses found that vector control activities had increased significantly since 2005, with a three-fold increase in ITN ownership and IRS coverage. However, coverage varied dramatically across the 17 countries; some regions reported >70% ITNs ownership and regular IRS activity, while others had no coverage in remote rural populations where the risk of LF was potentially high and co-endemic with high risk *L.loa*.

**Conclusions:**

Despite many African countries being slow to initiate MDA for LF, the continued commitment and global financial support for NTDs, and the concurrent expansion of vector control activities for malaria, is promising. It is not beyond the capacity of GPELF to reach its target of global LF elimination by 2020, but monitoring and evaluating the impact of these activities over the next decade will be critical to its success.

## Background

The Global Programme to Eliminate Lymphatic Filariasis (GPELF) has made remarkable progress since its inception in 2000, and is hailed to be the most rapidly expanding global health programme in history [1,2]. Nearly all 39 endemic countries in the Americas, Eastern Mediterranean and Asia-Pacific regions have initiated or finished the World Health Organization (WHO) recommended mass drug administration (MDA) campaign to interrupt transmission of the two main parasites, *Wuchereria bancrofti* and/or *Brugia malayi* with significant scale-up in drug distributions and reductions in disease burden being demonstrated [1,2].

This progress is promising for GPELF and its goal of LF elimination by 2020 [1]. However, for all countries to fully benefit from this global effort, it must address the slow progress in Africa where half of the 34 endemic countries have not or have only just started to implement the GPELF intervention strategy for LF elimination, which does not promote vector control, despite the suggestion of the need to link malaria and LF activities for mutual benefit [3-5], and the widespread evidence of the value of vector control in the control/elimination of LF [6,7].

The African continent has a large burden of LF, which is caused by the parasite *W. bancrofti* and predominantly transmitted by *Anopheles* mosquitoes in rural areas and by *Culex* in urban and coastal areas of East Africa [7-12]. While many countries have started MDA and made steady progress over the past decade, at the half way mark of GPELF in 2010 [1,13], there were still 17 endemic countries that had not started MDA activities for LF elimination. These countries included Angola, Central Africa Republic (CAR), Chad, Congo, Democratic Republic of Congo (DRC), Equatorial Guinea, Eritrea, Gabon, The Gambia, Guinea, Guinea-Bissau, Liberia, São Tomé and Príncipe, Zambia, Zimbabwe and the new Sudan/South Sudan [1].

There are several factors that have potentially impacted on the progress of these countries and their ability to fully establish their LF Programme, and launch or scale-up MDA activities. Many of these countries are among the poorest in the world according to the United Nations Human Development Index (HDI), with over half the countries among the 20 least developed countries in the world (i.e. CAR, Chad, DRC, Eritrea, The Gambia, Guinea, Guinea-Bissau, Liberia, Sudan/South Sudan, Zimbabwe) [14]. Many are conflict or post-conflict countries with fragile health systems, inadequate transport infrastructure, populations which are difficult to access and with a high number of internally displaced people [14,15]. Additionally, half of the countries are co-endemic with *Loa loa,* another filarial parasite that can cause severe adverse events (SAEs) after treatment with ivermectin [16-18]. This is a significant impediment for countries in West and Central Africa, particularly in DRC where both diseases are considered to be highly endemic [19].

These challenges pose major barriers for national LF programmes. Whilst twice yearly treatment of albendazole alone has been recommended as an alternative drug regime in LF/*L. loa* co-endemic areas where ivermectin cannot be safely used on a mass scale [20], it is imperative that additional strategies such as vector control, and links with large scale malaria programmes are considered, especially as interventions such as insecticide treated and/or long lasting insecticidal nets (ITNs/LLINs) [21] and indoor residual spraying (IRS) [22] for malaria control, have shown to impact LF in a variety of ecological settings where *Anopheles* are the primary vectors [23-31]. WHO is advocating for integrated vector management (IVM), encouraging better linkages between LF and malaria programmes [32], and has recently developed provisional strategies for interrupting LF in loiasis-endemic areas which involves vector control [18].

The objective of this study was to examine the magnitude and geographical extent of recent deployment of ITNs/ LLINs and IRS activities in 17 African countries to determine the potential additive impact of vector control interventions on LF and their potential contribution to the global goal of LF elimination by 2020.

## Methods

### Data sources

National data on the population at risk of LF and the *L. loa* co-endemicity status was obtained from the WHO Progress Report [1] for Angola, CAR, Chad, Congo, DRC, Eritrea, Equatorial Guinea, Gabon, The Gambia, Guinea, Guinea Bissau, Liberia, São Tomé and Príncipe, Sudan/South Sudan, Zambia, and Zimbabwe. The African map of LF developed by Lindsay and Thomas [7] was used to highlight the distribution of microfilaria (Mf) prevalence levels of 20-40%, and >40% across the continent, specifically in the 17 countries and in relation to *L. Loa* prevalence. The recent loiasis risk map for Africa defined from extensive field surveys of eye worm history [16,33,34], was used to highlight the distribution of *L.loa* prevalence levels of 20-40%, and >40% including the risk of SAEs. The LF and *L. Loa* maps were imported and digitised in the geographical information system software ArcGIS 10 (ESRI, Redlands, CA) to define the specific prevalence distributions, examine the extent of geographical overlap and to identify areas that would benefit from vector control.

National and sub-national data collected between 2000 and 2010 with specific geographical information on mosquito bed nets, ITNs and IRS were obtained from published literature, national reports, surveys and datasets from public sources including the WHO, Demographic Health Surveys (DHS), Malaria Indicator Surveys (MIS), and Multiple Indicator Cluster Surveys (MICS), World Malaria Reports, Roll Back Malaria (RBM) and President’s Malaria Initiative (PMI) websites [35-41]. Specifically, household-level data from DHS and MIS cluster surveys [38,39] with information on the geographical coordinates of each cluster (i.e. geo-referenced), were obtained to examine the finer spatial distribution of vector control interventions for malaria relating to household ownership and number of mosquito bed nets, and presence of IRS activities.

### National and sub-national analyses

National data on the percentage of households owning at least one ITN and percentage of IRS coverage between 2000 and 2010 were summarised [35,36]. The differences between urban and rural populations and the percentage of households with at least one type of mosquito bed net, at least one ITN and the percentage of children <5 years of age who had slept under any net the night prior to the survey were summarised and tabulated for each country based on all DHS, MIS and MICS reports available [38,39]. Sub-national data were based on recent provincial/state reports and survey data available from 2005 and used to create maps using software ArcGIS 10 (ESRI, Redlands, CA). The percentage of households with very low (<25%), low (25-50%), medium (50-75%) and high (>75%) coverage of at least one bed net (any type), at least one ITN and the presence of IRS activities were highlighted for each country with the data available. DHS geo-referenced cluster data (latitude and longitude coordinates) in geographically defined areas, with information on vector control activities were also used to map the spatial distributions of bed nets (any type) and IRS activities on a finer scale.

## Results

### National data analysis

**Figure 1:**
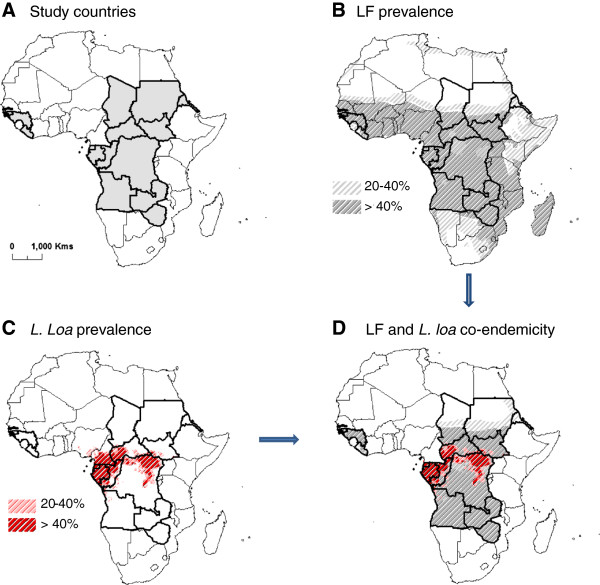
**LF and *Loa loa *prevalence and the extent of co-endemicity in 17 countries. A.** Study countries. **B.** LF prevalence. **C. ***L. loa* prevalence. **D.** LF and *L. loa* co-endemicity.

The estimated population at risk of LF is summarised in Table 1. Collectively these countries account for more than 100 million people at risk, representing approximately half of the estimated burden across Africa. Geographically, these countries cover vast, often remote, areas of the continent over more than 10 million km^2^ as shown in Figure 1a. The geographical limits of LF mapped in Figure 1b highlight the widespread nature of the disease with most of the 17 countries highly endemic with Mf prevalence estimates >40%. The distribution of *L. loa* was mainly found in Central and West Africa (Figure 1c). In total 8 countries are considered to be co-endemic with *W. bancrofti* and *L.loa* with the greatest risk of *L. loa* and SAEs in CAR, Congo, DRC, Equatorial Guinea, Gabon and the new country of South Sudan (Figure 1d).

**Table 1:** Summary of disease data and main vector control interventions 2000 to 2010

**Country**	**Risk of LF**	***Loa loa *endemic**	**Households (%) at least one ITN**		**IRS coverage (%)**	
			**2000-04 2005-10**		**2000-04 2005-10**	
**Angola**	12090000	Yes	6.0	17.7	0.2	3.6
**CAR**	3300000	Yes	4.6	19.7	-	-
**Chad**	7270000	Yes	5.2	7.2	-	-
**Congo**	2600000	Yes	1.8	7.7	-	-
**DRC**	49140000	Yes	2.8	26.5	-	0.1
**Equat. Guinea**	420000	Yes	2.2	30.0	8.0	32.2
**Eritrea**	3577000	No	58.2	68.7	4.5	4.6
**Gabon**	1290600	Yes	1.8	41.0	-	-
**Gambia**	1200000	No	24.2	43.2	-	35.1
**Guinea**	6067135	No	1.0	6.2	-	0.4
**Guinea-Bissau**	1311741	No	14.6	41.2	-	-
**Liberia**	3600000	No	1.8	37.8	-	-
STP*	410000	No	29.4	59.0	-	74.3
**Sudan**	-	Yes	1.8	17.7	6.9	6.7
**Zambia**	8780000	No	11.2	59.0	4.4	32.0
**Zimbabwe**	6000000	No	2.8	35.3	17.3	31.1

*STP=São Tomé and Príncipe.

**Figure 2:**
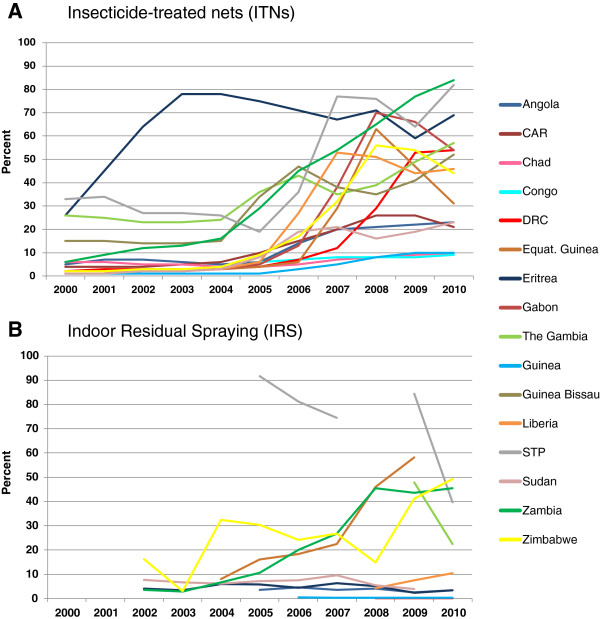
**National summaries of the proportion of households with vector control. A.** Insecticide-treated nets (ITNs). **B.** Indoor Residual Spraying (IRS). Note: Data obtained from the Malaria Report 2010, Chapter 4: Vector Control, Table 4.3 and Annex 5.

Data collected from the recent World Malaria Reports [35,36] estimate that the proportion of households owning at least one ITN (based on modeled estimates between 2000 and 2010), had increased three-fold overall since 2005 (Table 1), however, there was great variation between the 17 countries as highlighted in Figure 2a. Between 2000 and 2004, the average national ITN coverage was 10.6% for all countries and ranged from 1.0 to 58.2%, with the lowest coverage in Guinea 1.0%, Congo, Liberia and Sudan 2%, and the highest in The Gambia 24%, São Tomé and Príncipe 29% and Eritrea 58%. Data from between 2005 and 2010, showed the average national ITN coverage was 32.4% for all countries with a range from 6.2 to 68.7%, with the lowest coverages in Guinea 6.2%, Congo 7.7% and Chad 7.2% and the highest in Eritrea 68.7%, São Tomé and Príncipe 59%, Zambia 59% (Table 1, Figure 2a).

Similarly for IRS, the data available in the World Malaria Reports indicate that the proportion of people coverage by IRS differed over time and between countries as shown in Table 1[35,36]. Overall, there were fewer countries receiving IRS and the coverage was generally low. Between 2000 and 2004, the average national coverage was 6.9% for the six countries reporting this activity, and coverage ranged from 0.2% in Angola to 17.3% in Zambia (Figure 2b). This compared to data between 2005 and 2010, when the average national IRS coverage was 20.7% for the 10 countries reporting this activity, and ranged from 0.1% in DRC to 74.3% in São Tomé and Príncipe.

### National urban and rural comparisons

DHS, MIS [38] and MICS [39] reports indicate that differences between urban and rural populations in relation to the percentage of households with at least one bed net (any type), an ITN and children <5 years of age who had slept under any net are shown in Table 2. Overall, the highest coverage rates were found in urban areas except in selected surveys of Angola, Equatorial Guinea, Eritrea, Liberia and Zambia. Similar to data presented in Table 1, coverage rates were generally lower for the period 2000 to 2004 compared with 2005 to 2010. Overall, the majority of countries with data from two different time periods had a two- to three-fold increase in coverage over a 5 to 7 year period.

**Table 2:** Urban and rural comparisons of mosquito bed nets and ITNs ownership and usage

**Country**	**Year**	**Households (%) with at least one type of bed net**		**Households (%) with at least one ITN**		**Children (%) < 5 years slept under any bed net**	
		**Urban**	**Rural**	**Urban**	**Rural**	**Urban**	**Rural**
**Angola**	2000	-	-	-	-	10.8	9.6
**Angola**	2006	34.0	31.3	29.1	25.9	19.2	22.0
**Angola**	2011	41.5	33.5	39.0	31.8	30.7	25.2
**CAR**	2000	-	-	-	-	48.2	19.8
**CAR**	2006	54.0	25.9	26.1	11.2	52.4	21.7
**Chad**	2004	-	-	-	-	77.2	61.0
**Chad**	2006	-	-	-	-	57.5	18.6
**Congo**	2005	82.0	68.3	8.0	8.0	76.8	59.7
**Congo**	2011	86.4	79.6	18.9	39.7	83.8	76.8
**DRC**	2001	-	-	-	-	15.0	10.3
**DRC**	2007	37.8	21.5	12.4	7.1	26.0	14.3
**Equat. Guinea**	-	-	-	-	-	-	-
**Eritrea**	2002	28.3	37.3	-	-	14.3	11.0
**Gabon**	2000	-	-	-	-	-	-
**The Gambia**	2000	-	-	-	-	45.4	35.8
**The Gambia**	2005	48.5	69.6	34.0	64.0	54.6	67.6
**Guinea**	2005	27.5	26.5	1.0	0.3	15.7	11.0
**Guinea-Bissau**	2000	-	-	-	-	74.7	63.4
**Guinea-Bissau**	2006	81.5	77.8	34.5	48.9	79.9	70.5
**Liberia**	2007	31.3	29.9	-	-	-	-
**Liberia**	2009	44.5	52.7	42.0	51.8	25.6	28.2
**STP**	2000	-	-	-	-	60.4	27.1
**STP**	2006	58.4	37.3	44.1	25.4	61.8	41.0
**STP**	2008	75.2	61.1	69.2	52.4	72.9	52.3
**Sudan**	2000	-	-	-	-	25.8	20.6
**Zambia**	1999	-	-	-	-	8.5	4.7
**Zambia**	2001	34.9	23.4	16.1	12.4	21.9	13.7
**Zambia**	2007	64.3	64.4	52.6	53.7	35.0	32.8
**Zambia**	2008	66.2	73.6	58.8	63.9	42.8	49.3
**Zimbabwe**	2005	34.4	12.8	11.0	7.2	16.1	3.2
**Zimbabwe**	2010	46.9	38.2	23.2	31.6	18.8	11.6

Note: Data are based on DHS, MIS, MIC national survey reports available between 2000 and 2010.

For the period 2000 to 2004, there were limited data available on the percentage of bed nets (any type) and/or ITNs [38,39], however, there were 10 surveys reporting the percentage of children <5 years of age who had slept under any net, highlighting that the lowest coverage was in Angola, Eritrea, DRC and Zambia (urban 10.8- 15.0%; rural 4.7-11.0%) and the highest coverage in Guinea Bissau, São Tomé and Príncipe and Chad (urban 57.5 - 74.7%; rural 18.6 - 63.4%) (Table 2). The most significant increases in coverage after this period were the percentages of children using bed nets in rural areas of The Gambia (35.8% to 67%), in São Tomé and Príncipe (28.2% to 52.3%) and Zambia (4.7% to 49.3%).

For the period 2005 to 2011, countries with the lowest coverage of bed nets (any type) included Zimbabwe and Guinea (urban 27.5 - 34.4%; rural 12.8 - 26.5%) with the highest in Congo and Guinea Bissau (urban 81.5 - 82%; rural 68.2 - 77.8%) [38,39]. For ITNs, the lowest coverage was in the Congo, Guinea and Zimbabwe (urban 1.0 - 11%; rural 0.3 - 8.0%), and the highest in São Tomé and Príncipe and Zambia (urban 52.6 - 69.2%; rural 52.4 - 53.7%). Similarly, for children < 5 years of age who had slept under a bed net [any type], the lowest coverage was in Angola, Guinea and Zimbabwe (urban 15.7 - 19.2%; rural 3.2 - 22%) and the highest in Guinea Bissau, The Gambia, and São Tomé and Príncipe (urban 54.6-79.9%; rural 67.6-70.5%).

### Sub-national mosquito bed net and ITN maps

**Figure 3:**
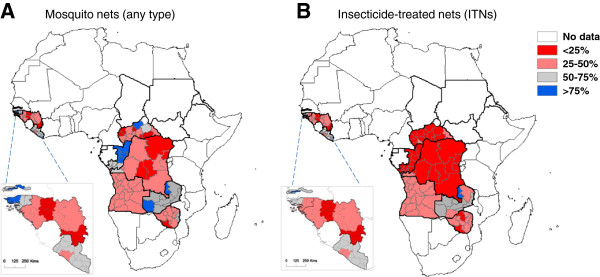
**Sub-national distribution of households in possession of at least one bed net and at least one insecticide-treated net (ITN). A.** mosquito nets (any type). **B.** Insecticide-treated nets (ITN). Note: Maps based on data from the following survey reports in Angola (MIS 2006–07), CAR (MICS 2006), Congo (DHS 2005), DRC (DHS 2007), The Gambia (MICS 2005–06), Guinea (DHS 2005), Guinea Bissau (MICS 2006), Liberia (MIS 2009), São Tomé and Príncipe (DHS 2008–09), Zambia (MICS 2008) and Zimbabwe (DHS 2005–06).

Maps of provincial and/or state level coverage were created from the report data [38,39] to highlight the broad distribution of bed nets (any type) and ITNs across Angola, CAR, Congo, DRC, The Gambia, Guinea, Guinea Bissau, Liberia, São Tomé and Príncipe, Zambia and Zimbabwe from survey data obtained between 2005 and 2011 (Figure 3a,b). No recent provincial and/or state level data were available from these data sources for Chad, Equatorial Guinea, Eritrea, Gabon or Sudan.

Overall, higher percentages were found across Congo, Guinea Bissau, The Gambia, Liberia, São Tomé and Príncipe and Zambia (Figure 3a,b). However, there were some differences between countries, for example, Congo had a higher percentage of bed nets (any type) than all other countries but very low ITNs, whereas Zambia had a relatively high percentage of both interventions (Figure 3a, b). In high risk *L.loa* countries (Figure 1), there was generally no information available or predominately low percentages of bed nets (any type) and ITNs reported, except in Congo where there was a very high (>75%) percentage of bed net use (any type).

**Figure 4:**
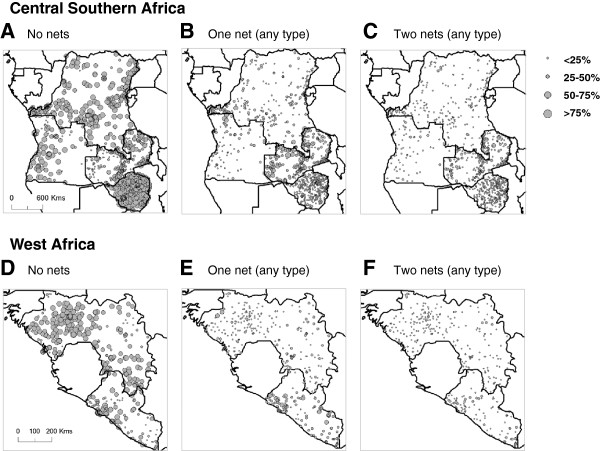
**Proportion of households per cluster with bed nets (any type).** Central Southern Africa: **A.** No net. **B.** One net. **C.** Two nets. West Africa: **D.** No net. **E.** One net **F.** Two nets.

Geo-referenced cluster data with information on the percentage of households with no bed net, one bed net (any type) and two bed nets (any type) were available for six countries including, Angola, DRC, Guinea, Liberia, Zambia, and Zimbabwe [38]. The number of clusters differed for each country, however, the overall trends were similar with a high percentage (>50%) of households reporting no ownership of a bed net (Figure 4a, d). There was a lower percentage (<25%) of households with one bed net, however, Liberia and Zambia reported higher coverages than the other countries (Figure 4b, e). Very few households in each cluster had two bed nets, with Zambia reporting the highest coverage (Figure 4c, f).

### Sub-national IRS data and maps

Information on IRS activities since 2005 were available for ten countries, including Angola, DRC, Equatorial Guinea, Eritrea, The Gambia, Guinea, Liberia, São Tomé and Príncipe, Zambia, and Zimbabwe. The main sources were WHO, RBM and PMI reports [37,40,41], and DHS reports and geo-referenced cluster data [38], with little or no information available from other sources. There was great variability in the detail of reporting including the population covered, geographical extent of activities and insecticides used, which mainly included pyrethroids (lambdacyhalothrin, alphacypermethrin), and/or organochlorines (DDT), however, carbamates were also reported in some countries where insecticide resistance had emerged in key vector *Anopheles* species [41].

**Figure 5:**
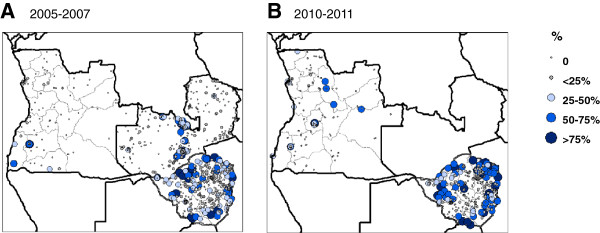
**Percentage of households reporting indoor residual spraying (IRS) activities. A.** 2005–2007. **B.** 2010–2011. Notes: Maps based on geo-referenced cluster data collected in Angola (2007, 2011), Zambia (2007), and Zimbabwe (2005, 2010).

Overall, the most extensive IRS activities were reported in Angola, Zambia and Zimbabwe. In Angola, IRS with lambdacyhalothrin was used periodically in selected areas of central and southern provinces since 2006. In Zambia, IRS activities were expanded rapidly from 2004 to reach full geographical coverage using different insecticides in different regions including lambdacyhalothrin, DDT, carbamates, and etofenpox. Similarly, Zimbabwe scaled up IRS activities significantly over the past decade using DDT and lambdacyhalothrin. The DHS geo-referenced cluster data on the percentage of households reporting IRS activities were available for all three countries for the period 2005–2007, and for Angola and Zimbabwe for the years 2010–2011 (Figure 5a, b). Comparison between the different time periods indicated an increase in IRS activities in Angola with six clusters from four provinces reporting >25% IRS activities in 2007, compared with 28 clusters from eight provinces in 2011. Zimbabwe had higher coverage overall, and 80 clusters from nine provinces reported >25% IRS in 2005, compared with 95 clusters in 2010. In Zambia, 69 clusters from nine provinces reported >25% IRS in 2005, however, no DHS cluster data were available thereafter for comparison.

The island nation of São Tomé and Príncipe, and Bioko Island of Equatorial Guinea also reported widespread IRS activities. In São Tomé and Príncipe, an IVM programme was initiated by the Ministry in 2003 which included IRS, space spraying and larviciding. Between 2005–2007, all districts were targeted with IRS using alphacypermethrin once a year with a reported coverage of 94% of households. No detailed reports are available, however, large-scale IRS activities were expected to continue until 2009. In Equatorial Guinea, IRS activities have been focused in Bioko Island covering all districts since 2004 using by deltamethrin and/or the carbamate, bendiocarb with 72% to 84% operational coverage reported.

IRS activities in the other countries report less in terms of geographical coverage and duration, with little data or detailed information available in the public domain. In DRC, IRS has been limited to heath zones where mining companies (extractive industries) are operating, mostly in the Katanga Province. In Sudan, IRS was carried out in targeted irrigated areas and in the Northern, River Nile and Kassala States. In Eritrea, IRS has been implemented in most malarious areas since 2004, initially with blanket coverage and then in selective areas using DDT and the organophosphate, malathion. In 2007, IRS was reported in nine districts across three zones of the country covering an estimated 8% of the population.

In West Africa, Liberia reported IRS activities in 2007–2008 in camps for internally displaced persons and refugees, and since 2010 IRS activities have been carried out in selected counties using DDT and pyrethroids. In Guinea, a survey in 2010 reported 8% of households had received IRS in the previous 12 months, with the Conakry Region reporting the highest coverage of 19%, while other regions with more than 2% IRS coverage included the mining areas of Boke, Kankan and Faranah. In The Gambia, a news article highlighted that IRS was introduced in the country in 2008 and implemented by local communities in 2009, and specifically launched in Janjangbureh, Central River Region in 2011, however, no further details or reports are available.

## Discussion

This study provides a broad overview of vector control activities in the 17 African countries that are yet to start, or have only recently started MDA implementation for LF elimination. Overall, the significant scale up of ITNs, and to a lesser extent IRS, is promising, and it is likely that these interventions that reduce malaria transmission [21,22,42-44], have also already impacted on the transmission of *W. bancrofti* in many co-endemic areas. However, monitoring their impact will be critical, and it may be more efficient and cost effective for the new LF Programmes to develop formal links with national malaria control programmes and include IVM as one of their key intervention strategies [32]. A recent review has highlighted several examples of IVM carried out in a range of ecological settings [9], which suggests that this integrated approach could be successful in Africa where the main vectors of malaria and LF are similar *Anopheles* species, and transmission may coincide [10,11,45-48]. In order to achieve this, it is critical that countries take advantage of the current funding available for malaria, as resources may be declining and the window of opportunity for vector control may be closing in the near future.

The reason for the variation in vector control coverage across the different countries may be due to a number of factors including the geographical size (e.g. São Tomé and Príncipe - small islands), and the relative wealth and political stability of a country (e.g. DRC, Guinea, Angola, Zimbabwe - post conflict) [14]. It may also be related to whether a country has an established and active malaria control programme (e.g. The Gambia - traditional high net coverage, Eritrea - high bed net coverage) [49-53], or has received substantial funding for malaria control from the government and international donors and particularly the Global Fund (e.g. Zambia - significant support from World Bank) [35,36]. These factors are complex and interacting, and how the GPELF can use this information to take full advantage of the increasing scale up of ITN and IRS activities, needs to be considered in the context of the LF endemicity and programmatic capacity of each country, as it can vary considerably [1,13].

It is possible that a number countries such as The Gambia, Eritrea and São Tomé and Príncipe may not need to develop a LF Elimination Programme or implement MDA at all, as they are small, politically stable, have established active malaria control programmes, relatively high bed net coverage and shown limited or no evidence of LF transmission in the past decade. While The Gambia has historical evidence of high LF transmission [54-56], it also has a long history of large scale bed net distribution [52,53], and recent reports suggest that LF is no longer a public health problem (unpublished data). Eritrea has little historical evidence [8,56], has no current data on LF, as programmatic mapping has not started [1], but has a successful vector control programme [49-51]. In São Tomé and Príncipe, historical reports indicate disease presence [56], and recent IVM activities are likely to have impacted transmission [35-41,49]. Therefore, it may be more appropriate to assess these countries for the interruption of transmission using the new Transmission Assessment Survey (TAS) developed by WHO [57,58]. Recently, WHO reassessed nine other countries and compiled sufficient evidence to reclassify them as non-endemic, thereby reducing the global number of endemic countries to 73 [59]. A similar assessment using TAS as a tool to verify the lack of transmission in The Gambia, Eritrea and São Tomé and Príncipe may help ‘shrink the map’, as well as contribute to considerable cost savings. Similarly, on Bioko Island, Equatorial Guinea the use of ivermectin in onchocerciasis control together with a high net and IRS use will have been likely to have an additive impact [60].

Zambia is another country with a well established vector control programme with relatively high bed net coverage, and some IRS activities, which also could have impacted on LF transmission [37,41,49,61,62]. However, in Zambia the LF Programme recently finalised baseline mapping and found two thirds of the population at risk of LF. Overall antigen prevalence rates were low ≤10%, except in a few selected regions of the country (unpublished data). The first MDA is due to start in early 2013 and together with the ongoing vector control activities, the LF Programme could see a significant reduction in transmission in most regions of the country within a few years. The only concern to note is that there is already widespread insecticide resistance found in several *Anopheles* species across the country and different strategies to overcome this are currently being developed [41,63].

The extent to which insecticide resistance prevents ITNs and IRS from being effective is not known, especially in relation to LF transmission. However, given the increasing geographical spread of resistance to multiple classes of insecticide across Africa [64-68], there seems to be another small window of opportunity to maximise the full potential of these alternative strategies. Alarmingly, almost all of the 17 countries have already reported some degree of insecticide resistance to one of the main classes of insecticides, in one of the main *Anopheles* species responsible for malaria transmission. The scope of this problem is yet to be fully determined, however, resistance monitoring is becoming increasingly important among international stakeholders such as PMI [41], and the development of publicly accessible up-to-date databases and maps, including the new IR Mapper [67,68], will help to assess the situation over time and space along with other programmatic activities [69-71]. This is particularly important given the limited availability of vector control tools and the fact that new products will not be available for some years [72]. It is also not know what effect insecticide resistance will have on filarial worm development in *Anopheles* mosquitoes, as in *Culex*, highly elevated esterases involved in insecticide resistance were found to inhibit development of microfilariae of *W.bancrofti* in a study in Sri Lanka [73].

The countries facing the greatest challenges are those that have endured conflict and civil unrest, and have among the lowest coverage of vector control interventions such as Congo, Guinea, and DRC [15,35-41]. New LF programmes in these countries are establishing themselves, with significant barriers related to the lack of public infrastructure, transport networks and trained health personnel. Malaria control programmes face similar constraints despite significantly more funding available for vector control [35,36,40,41]. In large countries such as DRC, significant geographical factors have been found to contribute to low bed net coverage in remote, rural areas [15], which suggests that alternative distribution methods are needed, especially where LF may be co-endemic with *L. loa* and ITNs are one of the main recommended strategies by WHO [20]. Increased efforts to boost vector control coverage in high risk *L. loa* co-endemic areas is critical, especially as potential alternative strategies such as twice a year albendazole have to go to scale whilst the use of doxycycline an anti-*Wolbachia* macrofilaricide or adult sterilising agent [74-76], has yet to be recommended or available for distribution without medical supervision.

The use of the established and extensive network of community drug distributors working for the African Programme for Onchocerciasis Control (APOC) in eight of the 17 countries may be an entry point to increase ITN distributions as has been demonstrated in Nigeria [77,78]. This will benefit both the LF and malaria programmes, and provide new opportunities for onchocerciasis programmes which may be scaling down due to their success as they move towards the elimination of transmission of *Onchocerca volvulus*[79,80]. Already many LF Elimination Programmes across Africa use the community-directed treatment with ivermectin (CDTi) strategy developed by APOC, as a platform to distribute MDA [1]. It seems sensible to extend this to include ITNs and other interventions in the remote hard-to-reach, co-endemic areas where CDTi already operates [78,81]. Finer scale mapping, using the micro-stratification overlap mapping (MOM) approach, could be used to identify the populations most at risk, so that ITNs distributions can be specifically targeted [19]. However, more collaboration, communication and coordination between the various NTD and malaria vector-borne disease control programmes is important and becoming an international priority.

## Conclusions

This study highlights that although many African countries are behind with initiating MDA, and populations remain at risk, the continued global commitment and financial support and the expansion of insecticide-based vector control activities, is promising. The scale-up of these interventions is unprecedented and provides a unique opportunity to impact significantly on two major vector-borne diseases in Africa. It is not beyond the scope of GPELF in reaching its target of global elimination by 2020, however, more evidence-based data are needed to firmly establish the association between malaria vector control activities and the decline in the indicators of transmission of LF, and monitoring and evaluating the impact of these activities over the next decade will be critical to its success.

## Competing interests

The authors declare that they have no competing interests.

## Authors’ contributions

LKH and MJB conceived the idea for the study. LKH identified data sources, developed the study design and collated, mapped and analysed the data. DHM and MJB contributed to the interpretation of results. LKH wrote the first draft, and all authors contributed to and read the final version of the manuscript.
